# Machine learning in mental health promotion for older adults: a scoping review

**DOI:** 10.1186/s12877-026-07543-2

**Published:** 2026-04-27

**Authors:** Yunchen Ruan, Haodong Liang, Seita Yamamoto, Shiqi Lin

**Affiliations:** 1 School of Humanities and Social Science, Fuzhou University, Minhou County, No. 2 Wulong Jiangbei Avenue, Fuzhou, Fujian 350108 China; 2 Department of Sociology, Peking University, Haidian District, No. 5 Yiheyuan Road, Beijing, China

**Keywords:** Machine learning, Scoping review, Mental health, Older adults, Healthy aging

## Abstract

**Objectives:**

Owing to the rapidly aging global population, an increasing number of older adults are experiencing mental health problems. Although machine learning has shown a lot of potential for promoting mental health in this group, there are no scoping reviews in this field. This study aimed to provide an overview of the applications of machine learning in promoting the mental health of older adults and identify associated trends and challenges.

**Methods:**

A scoping review was conducted based on the framework by Arksey and O’Malley. Three electronic databases, including Web of Science, PubMed, and IEEE Xplore, were systematically searched from database inception to March 15, 2026. We included English-language studies on the use of machine learning to promote mental health among older adults. Relevant information was extracted, summarized, and analyzed.

**Results:**

A total of 144 articles were included in this review. Our review showed that the current research reveals diverse data and algorithms, with machine learning applications concentrated in two directions. One is the prediction of the risk of mental health problems, and the other is the detection and identification of mental health status. There are still challenges in methodology and application directions, although machine learning effectively helps address some limitations of existing research.

**Conclusions:**

Future research may consider improving data quality, implementing longitudinal designs more extensively, enhancing model interpretability, and broadening research on various mental health problems and intervention–effect prediction. These initiatives may strengthen the empirical basis for clinical judgment and public health policy.

**Supplementary Information:**

The online version contains supplementary material available at 10.1186/s12877-026-07543-2.

## Introduction

With the rapidly aging global population, health conditions among older adults have emerged as an important public health issue. By 2050, the number of individuals aged ≥ 65 years is projected to reach 1.5 billion, accounting for 16% of the global population [1]. The demographic transition is evident not only in the growing number of older adults but also in the significant increase in human life expectancy. However, as individuals age, their functional abilities change, requiring adjustments in physiological and psychological aspects [2]. Mental health problems, especially depression, are becoming more common among older adults, affecting more than one-third of the global older adults population [3]. Other mental health problems, such as anxiety and loneliness, need close attention. Research indicates anxiety prevalence of 16.5% [4] and loneliness prevalence of 27.6% among older adults [5]. Mental health problems among older adults are often highly comorbid, i.e., they tend to occur concurrently or sequentially rather than alone. For example, 10.8% of Chinese older adults exhibit symptoms of both anxiety and depression [6]. Similarly, a study of older adults in the United Kingdom found that loneliness was strongly associated with an elevated risk and severity of depression, with this effect lasting for up to 12 years [7].

Mental health problems not only directly affect psychological well-being but also significantly reduce the quality of life in older adults by negatively affecting their physical health. Depression is a significant risk factor for cardiovascular disease and related mortality in older adults [8], and it is closely related to cognitive impairment [9]. A South Korean study found that anxiety symptoms in older adults were associated with an increased risk of heart disease and depressive symptoms were associated with a higher risk of asthma [10]. Furthermore, in China, loneliness was identified as a significant risk factor for the onset and deterioration of multiple chronic diseases [11]. Given this background, effective monitoring and improvement of the mental health status of older adults, as well as predicting potential risks, has emerged as a significant research priority in medicine, psychology, and demography.

As an important branch of artificial intelligence [12], machine learning (ML) can autonomously perform tasks by mimicking human learning processes. It uses algorithms to extract relevant information from existing data and continuously improves performance based on experience [13]. Deep learning (DL), a branch of ML [14], allows computational models that are composed of multiple processing layers to learn representations of data with multiple levels of abstraction [15]. ML has already had an extensive impact on empirical sciences [16]. Furthermore, it can explore and extract multiple explanatory variables from high-dimensional data and reveal complicated relationships between these variables. Considering that research on mental health in older adults frequently includes biological, social, and environmental factors, ML holds specific advantages and enormous potential in this field. It may find application in the analysis of various data types, including survey data, physiological indicators, and audiovisual materials, facilitating real-time monitoring and prediction of the mental health status and risks in older adults.

Existing studies do not provide a comprehensive overview of the available ML applications aimed at promoting mental health among older adults. This scoping review aimed to fill this critical gap by systematically reviewing studies published since 2012 to summarize the following aspects: 1) the trends and directions of ML applications in mental health promotion among older adults; 2) the challenges encountered in this field; and (3) potential directions for future research.

The following research questions were established to guide our analysis:

- Research Question 1 (RQ1): In research on the promotion of mental health in older adults, which data sources and types are utilized in ML and which algorithms are frequently used?
- Research Question 2 (RQ2): What are the application directions of ML in the field of mental health promotion in older adults?
- Research Question 3 (RQ3): What challenges does ML face in its application in promoting the mental health of older adults?

## Materials and methods

This review was conducted using a scoping review framework first outlined by Arksey and O’Malley [17] and later revised [18, 19]. Findings were reported in accordance with the Preferred Reporting Items for Systematic reviews and Meta-Analyses extension for scoping reviews (PRISMA-ScR) checklist [20]. To ensure research transparency, the Methods section follows the PRISMA-ScR guidelines and provides a detailed description of the search strategy, as well as the inclusion and exclusion criteria.

### Inclusion and exclusion criteria

According to the PCC framework, the inclusion criteria for this review were as follows: 1) Population (P): individuals aged ≥ 60 years (consistent with the United Nations’ definition of older adults [21]) or explicitly identified as older adults; 2) Concept (C): research on ML and mental health status in older adults, such as anxiety, depression, and loneliness; 3) Context (C): ML applications for promoting mental health in older adults, including prediction, monitoring, and intervention.

The exclusion criteria were as follows: 1) methodological studies that only introduced ML and unimplemented research protocols; 2) studies for which full texts were unavailable; 3) studies that did not specify the algorithms used or sample sources.

### Information source and study selection

This study utilized three major electronic databases—the Web of Science Core Collection, PubMed, and IEEE Xplore—as the primary sources for article retrieval. The search was conducted from the inception of each database to March 15, 2026. To maintain methodological rigor and the overall quality of the included studies, only peer-reviewed articles formally published in academic journals and written in English were considered. Grey literature databases were not included in the retrieval process.

The search strategy in this study was structured around three core concepts: machine learning, older populations, and mental health. A Boolean search strategy combining keywords and Medical Subject Headings (MeSH) terms was applied. The full search strategy is presented in the appendix (Table S1).

Two researchers independently assessed the studies based on the inclusion and exclusion criteria, following the initial search. In cases of a disagreement, a third researcher made the final decision. Notably, the Diagnostic and Statistical Manual of Mental Disorders (DSM-5) replaced the term "dementia" with "major neurocognitive disorder," classifying it under a distinct category of neurocognitive disorders to clearly differentiate it from psychiatric disorders such as depression [22]. Consequently, dementia and other organic diseases were excluded during the screening process.

### Data extraction

To systematically capture the key characteristics of each included article, a standardized table was developed. This table included author(s), location, publication year, aim, data source(s), sample size, ML algorithm(s) used, and model function. The standardized table was pilot-tested by two researchers on at least three included studies to ensure comprehensive and accurate data capture. Data extraction was then performed independently by the two researchers. Any discrepancies were resolved through discussion, with the senior author consulted when necessary.

## Results

### Search results

**Fig. 1:**
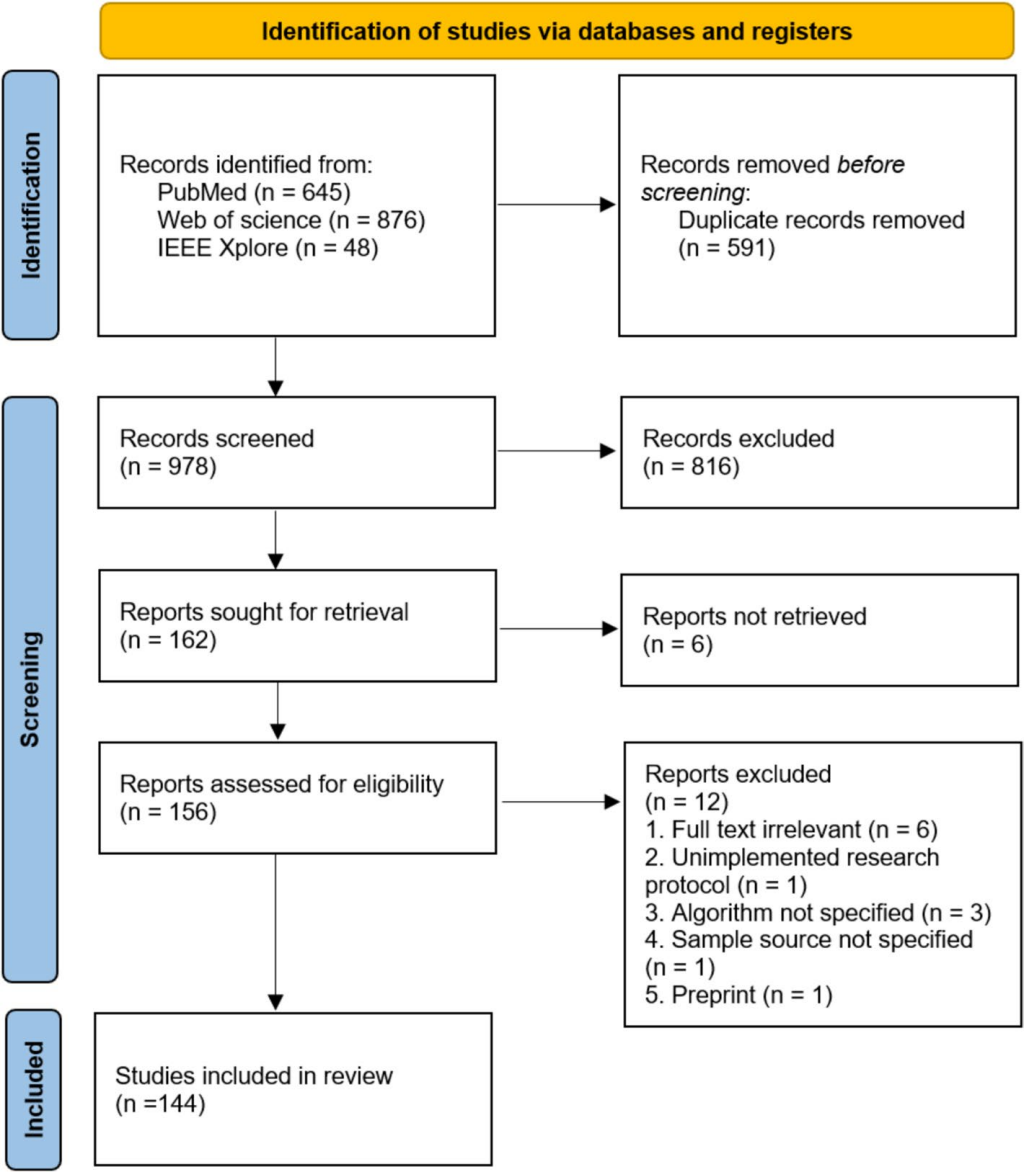
PRISMA flow chart of the selection process

After removing duplicates, a total of 978 records were initially identified. Following title and abstract screening and full-text evaluation, 144 studies were ultimately included for data extraction and integrated analysis [23–166]. Fig. 1 shows the article selection process.

### Results of data extraction

Most included studies were conducted in China (n = 76), the United States (n = 20), and South Korea (n = 20). All studies were published between 2012 and 2026, with the majority published after 2021. Table 1 lists the characteristics of some studies. Given the large number of studies included, Table 1 only presents a partial selection. The appendix (Table S2) provides the characteristics of all included studies.

**Table 1:** Summary characteristics of selected included studies

Authors (Year)	Location	Aim	Data Source(s)	Sample Size (Case)	Algorithm(s)	Model Function
Chen et al. (2026)[161]	China	Depression Prediction	Survey data of older adults in Guangzhou, China	2,585	LR, SVM, RF, XGBoost	Identifying predictors of depression in older adults
Alexopoulos et al. (2021)[78]	USA	Suicide Prediction	Recruitment by Weill Cornell Medicine and the University of Washington (UW) from 2014 to 2019	249	LASSO, RF, DT, and GBM	Identifying predictors of different suicidal ideation trajectories
Cruz et al. (2023)[100]	France	Well-being Prediction	Survey of Health, Ageing, and Retirement in Europe (SHARE)	37,991	RF	Ranking the importance of predictors of subjective well-being in the elderly
Chen et al. (2024)[102]	China	Life Satisfaction Prediction	2011–2018 China Health and Retirement Longitudinal Survey (CHARLS)	2,600	RF, XGBoost, SVM, kNN, and MLP	Identifying predictors of life satisfaction trajectories in the elderly
Xian et al. (2026)[142]	China	Anxiety Prediction	2018 Chinese Longitudinal Healthy Longevity Survey (CLHLS)	9,535	RF, XGBoost, LightGBM, DT, LR	Determining predictors of anxiety symptoms in older adults
Chen et al. (2025)[65]	USA	Depression Detection	Recruited elderly drivers	157	XGBoost, and LR	Identifying the depression status of the elderly through natural driving data
Maestro et al. (2023)[107]	Sweden	Emotion Detection	Physiological data collected by wearable medical sensors and data collected through a smartphone app with an EMA questionnaire	4	SVM, RF, and MLP	Automatically detecting and classifying the emotional states of the elderly
Castillo-Hornero et al. (2024)[34]	Spain	Loneliness Detection	Natural language texts collected via chatbots and online questionnaires on the Qualtrics platform	1,112	LR, and NB	Identifying the level of loneliness in the elderly from natural language
Finze et al. (2024)[62]	Germany	Well-being Detection	Recruitment from institutions where elderly individuals gather weekly for charity programs, mutual conversations, lectures, and games	32	SVM, LASSO, RF, and XGBoost	Assessing the subjective well-being of the elderly based on natural speech
Konig et al. (2021)[75]	France	Comprehensive Mental Health Detection	Recruitment through the Memory Clinic at Claude Pompidou Institute, Nice University Hospital	141	SVR, and LASSO	Extracting information from speech features to predict Neuropsychiatric Inventory (NPI) scores

*DT *Decision Tree, *GBM *Gradient Boosting Machine, *kNN *k-Nearest Neighbor*, LASSO *Least Absolute Shrinkage and Selection Operator*, LightGBM *Light Gradient Boosting Machine, *LR *Logistic Regression, *MLP* Multi-Layer Perceptron, *NB *Naive Bayes, *RF *Random Forest, *SVM *Support Vector Machine, *SVR *Support Vector Regression, *XGBoost *eXtreme Gradient Boosting

### Results of the included studies: a summary

#### Data and algorithms used

All included articles reported their data sources, with only two studies not specifying the sample size [51, 79]. The datasets used in these studies spanned multiple countries and regions, such as China, the United States, South Korea, and Europe. The data originated from various sources, such as large-scale surveys, regional or institutional field recruitment, online recruitment, special research projects, clinical trials, and open-access online databases. Large-scale survey datasets were exemplified by the China Health and Retirement Longitudinal Study (CHARLS), the Health and Retirement Study (HRS), and the Survey of Health, Ageing and Retirement in Europe (SHARE). These sources comprised a wide range of data types, including self-reported information collected through questionnaires, voice, image, text, facial expression, biomarker, and physiological or behavioral data captured by sensors. Several studies combined multiple data sources for analysis [24, 48, 59, 79, 94, 95, 101, 107, 113, 118, 135, 136]. The sample sizes ranged widely, from few individuals to tens of thousands, reflecting the diversity of research designs.

ML algorithms covering several fundamental and widely used categories were applied in studies aiming to promote the mental health of older adults. Most of the included studies used two or more algorithms (n = 103), thus allowing performance comparisons. The most commonly used ML algorithms in these studies were Random Forest (RF) (n = 100), Logistic Regression (LR) (n = 56), Support Vector Machine (SVM) (n = 53), eXtreme Gradient Boosting (XGBoost) (n = 50), and Decision Tree (DT) (n = 43), indicating that supervised learning was predominant. In addition, the use of stacking and ensemble methods was notable (n = 6). For example, a study developed five single prediction models and six stacked ensemble models to predict depressive disorders, with one stacked model achieving the best predictive performance [43]. In addition, two studies integrated Natural Language Processing (NLP) with ML algorithms, using NLP to extract structured features from text and then feeding these features into ML models to forecast specific psychological status [49, 88].

#### Application directions of ML

The applications of ML in the field of mental health promotion for older adults are focused on two directions: 1) developing predictive models to identify risk factors for mental health problems such as depression and anxiety, thereby providing a foundation for early intervention and individualized prevention and 2) utilizing various data and technologies to achieve real-time detection and identification of mental health status. Of the included studies, 122 focused on risk prediction and 21 focused on mental health status detection and identification. One additional study addressed both application directions [144]. In general, the distribution of application directions demonstrates a considerable imbalance in this field.

- (1) Developing predictive models to identify risk factors for mental health problems
  The topic distribution of research on predicting mental health problems in older adults was noticeably imbalanced, with the majority concentrating on depression prediction (n = 65). Additional mental health problems have been addressed, including suicidal thoughts (n = 13), well-being (n = 7), life satisfaction (n = 7), anxiety (n = 4), loneliness (n = 2), social isolation (n = 2), self-perceived aging (n = 2), quality of life (n = 2), psychological resilience (n = 2), self-neglect (n = 1), death anxiety (n = 1), death attitudes (n = 1), kinesiophobia (n = 1), self-compassion (n = 1), and comprehensive mental health status (n = 9). Notably, two studies addressed the prediction of two distinct mental health conditions simultaneously [116, 139]. These studies usually developed prediction models based on one or more combinations of environmental, socioeconomic, psychological, and physical health factors. Lin et al. examined the associations between loneliness and factors such as functional limitations, living conditions, environmental influences, age-related health issues, and health behaviors in older Chinese adults [46]. Meda et al. used random forest models to determine key predictors of suicide among variables such as demographics, physical health, depression, and cognitive function [69]. Using more predictive factors, especially considering more dimensions, will contribute to enhanced predictiveness and generalizability of ML models [167]. Based on this foundation, a research paradigm combining trajectory modeling with ML prediction has gradually emerged. The typical approach involves simulating the dynamic trajectories of mental states using longitudinal data and then applying ML algorithms to identify the factors determining different trajectory types. For example, Chen et al. used the latent class growth model and growth mixture model to classify distinct life satisfaction trajectories among older adults in China; afterward, they used ML algorithms such as random forest to determine key predictors for trajectory types [102]. This approach reflects a change away from static symptom prediction toward dynamic developmental prediction, providing new methods and evidence for the prevention of mental health problems in older adults.
  Most of the predictive research using ML targeted the general older adult population. Wang et al. identified depression risk factors among older Chinese adults using several ML algorithms [23]. Trescato et al. developed a model to forecast changes in subjective well-being of older adults in Europe over a 2-year period [27]. Perera et al. applied ML to determine the relative importance of five life domains in explaining overall life satisfaction among older adults in the United States [91]. Studies targeting diseased or hospitalized older adults focused on predicting disease-related mental health problems. Furthermore, recent research revealed that diseases or poor physical conditions can trigger or worsen mental health problems in older adults [168–170]. Zheng et al. used multiple kinds of ML algorithms to predict depression in older adults with chronic diseases [92]. Xiong et al. developed a prediction model for suicidal thoughts in hospitalized older adults with non-psychiatric conditions [37]. In addition, some studies focused on the mental health problems in specific older adult groups. Kim et al. developed a type of ML algorithm to predict the classification of depressive symptoms among older adults living alone in South Korea [39]. Chen et al. used double machine learning (DML) to identify the relationship between overage labor and depression among rural older adults, finding that overage labor significantly reduced depression levels in this group [80].
  Some studies have broadened the field of prediction to investigate the association between environmental elements and mental health, which is indicative of an interdisciplinary integration tendency. In addition to routine survey data, these studies used environmental data, such as street-view images. Additionally, some researchers examined the association between external environmental elements and the mental health of older adults, following the use of DL models to quantify these elements. For example, one study used a Fully Convolutional Network (FCN) to assess the quantity and quality of visual green space [59]. In another study, an FCN was utilized to conduct semantic segmentation of street-view images, allowing for the identification of 150 different object categories, such as the sky, buildings, and vegetation [95].
- (2) Detection and identification of mental health status
  Depression remains the primary focus of research on the detection and identification of mental health status in older adults. Six of these studies focused on detecting depression. Three studies detected both depression and anxiety, and one study simultaneously assessed loneliness and social isolation. Other studies focused on emotion (n = 3), loneliness (n = 3), well-being (n = 1), stress (n = 1), suicidal thoughts (n = 1), and comprehensive mental health status (n = 2). Additionally, data diversity has become a significant characteristic, with the use of voice data particularly notable. Lin et al. developed a model to screen and diagnose depression in older adults using voice data [24]; Finze et al. developed a model to assess the subjective well-being of older adults using natural speech data [62]. In addition to voice data, natural language text has been used to detect and identify mental health status. Badal et al. used NLP to detect social isolation and loneliness among older adults from interview transcripts [49]. Castillo-Hornero et al. identified levels of loneliness in older adults using natural language texts collected via chatbots and online questionnaires [34]. Additionally, facial expressions have been used to detect and identify the mental health status of older adults. Huang identified depression in older adults by extracting facial micro-expressions [51]. Zhou et al. developed a multi-class emotion classification model based on both linguistic and facial expression data that accurately differentiated emotions such as depression, anxiety, and apathy [42]. Furthermore, the physiological data provided by wearable devices enables real-time monitoring of emotions and stress levels in older adults. Maestro et al. used physiological data from wearable medical sensors to automatically detect and classify emotions in older adults [107]; Nath used wearable sensor data to measure stress levels in older adults [72]. Moreover, daily activity data have been used to help identify the mental health status of older adults. Lee et al. combined activity tracking data with geriatric assessment scale data to develop an ML method that detects depression and anxiety in older adults with mild cognitive impairment [63]. Chen et al. suggested a method for detecting depressive symptoms in older adults based on natural driving data [65].

#### Challenges in the application of ML

Based on the included studies, the use of ML in mental health promotion for older adults faces two challenges: methodology and application directions. Methodological challenges primarily concern data quality and research design. Data quality has a significant impact on the performance of ML models [171]. The efficacy of ML algorithms is highly dependent on large-scale, high-quality data; therefore, poor data quality or bias can reduce their accuracy [172]. The included studies used several large survey datasets, such as the CHARLS, NHANES, and SHARE. These datasets contain structured demographic, behavioral, and health indicator data with large sample sizes, and they are suitable for longitudinal analyses and causal inferences. However, such datasets may have several missing values for certain variables. Directly ignoring or excluding missing values may result in biased analysis results [173]. Besides, key variables may be missing the dataset, and insufficient variable coverage will affect the accuracy of the predictions by the models [99]. Additionally, these datasets have challenges related to data timeliness. Key predictors may have been altered significantly between the release of the data and subsequent research [92]. Self-reported questionnaire data—whether obtained from big datasets or small-scale surveys—are susceptible to subjectivity, resulting in departures from real characteristics [174] and differences between subjective and objective measurements [54]. Some studies with small sample sizes, particularly those focusing on detection and identification, have limitations such as insufficient data volume [107]. Limited data can easily lead to model overfitting [73, 175], resulting in inadequate conclusions [176].

In addition, there are challenges associated with the research design. First, for research lacking validation with external independent cohorts, the reliability and generalizability of the model need to be further tested [44]. Second, several studies used a single ML algorithm without comparing different algorithms, suggesting a potential for improved performance. Furthermore, although cross-sectional designs could provide valuable insights into associations between variables, they cannot define the direction of these relationships or confirm causal mechanisms [66]. Finally, the “black box” effect of ML remains a major obstacle to its broader adoption, particularly in healthcare [177]. Some have not adequately addressed model interpretability, resulting in a limited understanding of the factors driving model decisions, which may restrict clinical application and credibility [45].

The main challenges in application directions are the limited scope and imbalanced distribution of research topics. First, studies focusing on detection and identification remain scarce, although such research holds significant practical value in aging societies. As the risk of mental health problems among older adults increases, their likelihood of actively seeking professional help remains low [178]. Therefore, real-time detection and identification could provide critical opportunities for early intervention and personalized support, playing an important role in improving mental health outcomes for older adults. Second, the focus on mental health problems among older adults is significantly imbalanced. Existing studies primarily focus on depression, with limited exploration of other mental health problems such as anxiety, loneliness, and suicidal thoughts. This imbalance restricts the scope of ML applications in the field of mental health promotion for older adults. Finally, only three included studies predicted intervention effects on the mental health of older adults [73, 109, 159], although this research direction is crucial in aging societies.

### Future research directions

#### Methodology

Improving data quality should be the priority. ML-based imputation techniques, such as k-Nearest Neighbor (kNN) and sequential kNN, can effectively fill in missing values and diminish the negative effects of incomplete data on model performance [179]. To address the problem of missing key variables due to single-source data, future research could combine several data sources, such as public datasets, clinical indicators, and environmental exposure data, to improve the generalizability of predicted results [97]. Current research applying ML to mental health promotion for older adults is restricted in both depth and breadth of data integration, particularly regarding data diversity. The problem of data timeliness may be addressed in future studies by using longitudinal databases for model training, exploring new real-time data sources, such as physiological and behavioral data gathered by wearable sensors, and developing ML algorithms that can process data in real time. Because self-reported data are susceptible to subjective bias, studies that rely on these data should integrate them with objective measurements to reduce subjectivity-related errors or cross-validate them across other data types. Moreover, considering the small sample sizes in some research, large-scale validation research is still required [29]. When data size is inherently constrained, methods such as optimized feature selection, hyperparameter tuning, and ensemble modeling can be applied to reduce overfitting [73].

In addition, the research design requires further optimization. Studies lacking external independent cohort validation cannot adequately evaluate model generalizability, even when rigorous internal cross-validation is performed. Therefore, future research should include external validation using more diverse populations. Comparing the performance of multiple ML algorithms has become an emerging trend, and this strategy can be further leveraged to identify opportunities for model improvement. As cross-sectional designs do not allow for causal inference, the more extensive implementation of longitudinal designs in future research is strongly recommended. Two main approaches are used to improve the interpretability of ML. The first employs inherently interpretable models, often called “white-box” or “gray-box” models, such as decision trees and sparse linear models. Their simple structures and intuitive logic make them naturally understandable but they are limited in handling complex data [180]. The second approach uses post hoc interpretation methods such as Local Interpretable Model-agnostic Explanations (LIME) and Shapley Additive Explanations (SHAP). LIME fits local linear models to explain single prediction outcomes and is suitable for local analyses [181]; SHAP quantifies feature importance based on Shapley values, providing global and local interpretability [182].

#### Application directions

Regarding application directions, future studies should focus more on detecting and identifying mental health status. Such studies have tremendous practical utility and developmental potential, as they provide feasible ways for non-invasive mental health monitoring in community and home settings and enable earlier and more precise interventions in clinical practice. Furthermore, research should be expanded to focus more on mental health problems such as anxiety, loneliness, and suicidal thoughts to improve the overall mental health of older adults. Finally, using ML to predict the outcomes of mental health interventions is valuable, as it can provide scientific evidence to guide public health policy and optimize interventions in clinical practice.

## Discussion

### Principal findings

The rapidly aging population has made mental health problems in older adults an important issue across multiple disciplines. This scoping review summarizes the current applications and challenges of ML in the field of mental health promotion in older adults and explores potential future directions. Except for one study [81], all the included research was conducted within the past decade, indicating growing scholarly attention to this field in recent years.

First, the application of ML in mental health promotion among older adults shows considerable diversity in terms of data and algorithms. The included studies used a wide range of data sources, including large-scale survey datasets (such as CHARLS, NHANES, and SHARE), online or offline recruitment, specialized research projects, clinical trials, and open-access online databases. These sources covered various data types, such as self-reported information, voice, image, text, facial expression, biomarker, and sensor-based physiological and behavioral data. Integrating multi-source data has become a distinctive feature of current research. For example, some studies combined questionnaire data with street-view images [59, 94, 95, 101, 136] to examine the effect of environmental factors on the mental health of older adults. The integration of multi-source data not only improves data comprehensiveness, allowing models to capture more complex and subtle patterns of health change, but also broadens the application scope of ML, offering new opportunities for a better understanding of mental health in older adults. At the algorithmic level, the most commonly used algorithms include RF, LR, SVM, XGBoost, and DT, indicating that supervised learning dominates the field. Notably, the use of multiple algorithms for performance comparison has become a growing trend. Some studies have further applied model stacking and ensemble strategies to achieve performance optimization. In addition, a few studies have integrated NLP with traditional ML algorithms by first extracting linguistic features and then feeding them into models, such as RF, for detection or prediction [49, 88]. Although the number of such studies remains limited, they demonstrate the promising potential of ML for future applications.

Second, regarding application, existing studies primarily focused on two directions: 1) developing predictive models to identify risk factors for mental health problems and 2) detecting and identifying mental health status. Among these, depression received the most research attention. Comparatively, research on prediction yielded more results, whereas those on detection and identification remained at an exploratory phase. ML holds interdisciplinary potential in the field of mental health promotion among older adults. A few studies integrated theories and methods from psychology, medicine, computer science, urban planning, and sociology. One study used street-view data and DL models to quantify environmental features and examine their association with depression symptoms in older adults [101]; another study identified depression symptoms using plasma biomarkers [81].

Finally, this review identified several challenges in existing studies and suggests future directions. Methodologically, data quality has a significant impact on model performance. Despite their benefits of data scale and longitudinal tracking, large-scale survey datasets are susceptible to problems with missing values, inadequate variable coverage, and time lag. Self-reported information from questionnaires is prone to subjectivity, whereas studies with limited samples have the risk of model overfitting. Therefore, future research should more extensively consider using imputation techniques based on ML, integrating multi-source heterogeneous data, and leveraging long-term cohort datasets or immediate feedback devices to improve the timeliness and objectivity of model outputs, thereby mitigating the negative impact of data quality deficiencies on research findings and practical applications. Regarding research design, the lack of external independent cohort validation and cross-method comparison limits model reliability and generalizability. Additionally, cross-sectional designs cannot support causal inference. The robustness and interpretability of the model should be improved in future research by applying algorithmic comparisons, employing longitudinal designs, and using external validation in broader populations. Meanwhile, model interpretability remained a major challenge in ML applications. Adopting inherently interpretable models and post hoc interpretation methods, such as LIME and SHAP, could improve transparency and strengthen the clinical credibility of conclusions. At the application level, existing studies were too focused on prediction; research on detection and identification is insufficient; and there is a lack of exploration on non-depressive mental health problems such as anxiety, loneliness, and suicidal ideation. Future research should focus on detection and identification, as well as other mental health problems, and expand the application scope of ML in mental health of older adults. In addition, future studies may attempt to use ML to predict the prognostic effects of different mental health interventions and their heterogeneity in the population, thereby providing more scientific empirical evidence for the formulation of public health policies and clinical decision-making.

In summary, ML provides new technical support for mental health promotion among older adults. Therefore, it is necessary to further examine the specific contributions of ML in addressing the limitations faced by previous research. First, conventional generalized linear regression models are limited in handling nonlinear relationships, whereas ML can effectively address this issue, making it particularly suitable for analyzing large-scale data and exploring complex relationships among multiple variables [28]. Second, ML can reveal latent complex associations and patterns in data without requiring prior assumptions, while efficiently evaluating the contributions of different factors to health outcomes [97]. Third, in studies with small sample sizes, DL and SVM, when combined with discriminant analysis and data augmentation techniques, can help mitigate the constraints imposed by inadequate data [183]. Fourth, ML excels in processing high-dimensional data, enabling efficient feature selection and identification of key variables. For example, a study on late-life depression used SVM to identify three core plasma proteins from 195 plasma proteins [33]. Fifth, ML can handle multiple types of data, including voice, images, and text, significantly expanding the scope of research in mental health promotion for older adults. Sixth, ML can leverage devices such as wearable sensors to achieve real-time data collection and analysis, providing a feasible approach for continuous monitoring and early warning of mental health status in older adults.

### Geriatric-specific analysis

As a scoping review targeting older adults, this study examines the age-group-specific characteristics of ML applications in this population. Among the 144 included articles, 57 utilized longitudinal aging datasets, such as CHARLS, HRS, and SHARE. These datasets offer notable advantages in several research contexts: 1) multi-wave designs allow early-life characteristics to predict subsequent outcomes; 2) longitudinal data support long-term clustering, trajectory modeling, and predictor identification; 3) certain surveys share structural similarities in design (e.g., CHARLS was modeled after HRS, ELSA, and SHARE), enabling cross-cultural generalizability testing; 4) large sample sizes facilitate subgroup analyses based on extracted characteristics; and 5) these datasets can be integrated with external time-series data to broaden research scope.

However, compared with non-age-specific data sources, these datasets also have several limitations: 1) attrition due to survivorship bias is strongly correlated with study outcomes, potentially introducing systematic bias [104]; 2) self-reported mental health status among older adults is susceptible to recall bias, stigma, and personality traits [27, 52, 120], while proxy reports may exaggerate or understate the actual condition [69]; 3) cross-sectional measures make it difficult to distinguish transient symptom fluctuations from genuine change [126]; 4) early-life experiences can only be captured through retrospective self-report, precluding the estimation of full life-course trajectories [100]; 5) limited variable coverage may result in omitted variable bias [35]; and 6) despite design similarities across some surveys, the absence of identical measures and variables remains a barrier to external validation [92].

Furthermore, the included articles have not adequately addressed how age-group-specific characteristics may compromise the validity of data labels. This problem appears in two principal ways: 1) somatic symptoms associated with prevalent illnesses in older adults often overlap substantially with symptoms of mental health problems such as depression [184], potentially leading to systematic overestimation of scores on instruments such as the CES-D-10; and 2) older adults may tend to attribute negative emotions to physical discomfort rather than emotional distress [185], such that mental health problems are likely underestimated when assessment tools rely primarily on affective symptom items. Future studies encountering either limitation should explicitly acknowledge them.

Finally, older adults themselves represent a markedly heterogeneous population. The included articles have covered a range of subgroups, including older adults with chronic diseases, hospitalized patients, those with cognitive impairment or subjective cognitive decline, those living alone, those with functional limitations or disability, those with existing mental health problems, urban and rural residents, those who are still employed, and older drivers. Nevertheless, several subgroups remain understudied, including the oldest-old, sexual and gender minority older adults, older adults who have relocated across national borders, and those residing in long-term care facilities. Future research should prioritize these underrepresented subgroups to provide more targeted support for the broader older adult population.

### Limitations

This study also has several limitations. First, grey literature (e.g., dissertations and conference proceedings) was not included. However, as peer-reviewed journals are the primary source of high-quality evidence, the included studies still provide a comprehensive overview of research in this field. Second, only a limited number of electronic databases were searched, and only English-language studies were included, which may have led to the omission of relevant research. Third, no formal quality assessment was conducted, as scoping reviews primarily aim to provide an overview of existing evidence rather than evaluate study quality.

## Conclusions

This review systematically examined the applications of ML in the field of mental health promotion for older adults. The findings indicate that existing studies demonstrate diversity in data and algorithms, with two major directions of application: developing predictive models to identify risk factors for mental health problems and detecting and identifying mental health status. Although ML shows substantial potential for advancing mental health promotion among older adults, challenges remain in both methodology and application directions. Future studies should improve data quality, consider longitudinal research and external validation and emphasize model interpretability. In addition, researchers should explore a broader range of mental health problems and predict intervention effects, thereby providing a stronger scientific foundation for policy development and clinical practice related to the mental health of older adults.

## Supplementary Information

Supplementary Material 1.

Supplementary Material 2.

## Data Availability

All data generated or analysed during this study are included in this published article and its supplementary information files.

## Abbreviations

ML: Machine Learning
DL: Deep Learning
PRISMA-ScR: Preferred Reporting Items for Systematic Reviews and Meta-Analyses Extension for Scoping Reviews
MeSH: Medical Subject Headings
DSM-5: Diagnostic and Statistical Manual of Mental Disorders, 5th Edition
CHARLS: China Health and Retirement Longitudinal Study
HRS: Health and Retirement Study
SHARE: Survey of Health, Ageing and Retirement in Europe
NHANES: National Health and Nutrition Examination Survey
LIME: Local Interpretable Model-agnostic Explanations
SHAP: Shapley Additive Explanations
CES-D-10: Center for Epidemiologic Studies Depression Scale (10-item version)
