# Supplementary material for: Machine learning in mental health promotion for older adults: a scoping review
Source: BMC Geriatr. 2026 Apr 27;26:821. doi: 10.1186/s12877-026-07543-2 (PMC13255277; doi:10.1186/s12877-026-07543-2)
Supplement: Supplementary file 1 — Supplementary Material 1. [file 12877_2026_7543_MOESM1_ESM.docx]

**Table S1.** *The search strategy used in the electronic databases.*

| Database | PubMed, Web of Science, IEEE Xplore |
| --- | --- |
| Search strategy | #1: "machine learning" OR "deep learning"  #2: "aged" OR "elderly" OR "older adults" OR "population aging"  #3: "mental health" OR "well-being" OR "happiness" OR "satisfaction" OR "depression" OR "suicide" OR "loneliness" OR "social isolation" OR "sense of value" OR "meaning in life" OR "anxiety"  #4: "middle-aged" OR "midlife" OR "middle age" OR "adolescent"  #5: #1 AND #2 AND #3 NOT #4  All results were limited by language: English; publication date: from database inception to March 15, 2026. |

*Notes: Database-specific search restrictions were applied as follows: (1) Web of Science: Searches were limited to "Article" document types and queried using the "Topic" field. (2) PubMed: Queries were restricted to the "Title/Abstract" field, with filters appended to exclude "Conference Proceedings" and review articles (specifically "Review," "Systematic Review," "Meta-Analysis," and "Scoping Review"). (3) IEEE Xplore: The search was confined to "Journals," utilizing the "All Metadata" field for keyword retrieval.*

**Table S2.** *Summary characteristics of all included studies.*

| Authors (Year) | Location | Aim | Data Source(s) | Sample Size (Case) | Algorithm(s) | Model Function |
| --- | --- | --- | --- | --- | --- | --- |
| Alexopoulos et al. (2021) | USA | Suicide Prediction | Recruitment by Weill Cornell Medicine and the University of Washington (UW) from 2014 to 2019 | 249 | LASSO, RF, DT, and GBM. | Identifying predictors of different suicidal ideation trajectories |
| Andrews et al. (2017) | UK | Depression Prediction | Data collected to validate the NANA system | 40 | LASSO. | Generating predictive indicators of depression status based on emotional self-report data obtained by older adults via touchscreen |
| Arnold et al. (2012) | USA | Depression Prediction | A multiple biochemical biomarker study | 566 | RF. | Distinguishing the level of depressive symptoms in the elderly based on plasma biomarkers |
| Badal et al. (2021) | USA | Loneliness Detection  Social Isolation Detection | Elderly individuals living alone in a Continuing Care Senior Housing Community (CCSHC) in Southern California | 97 | ANN, SVM, kNN, DT, and RF. | Detecting social isolation and loneliness in the elderly with natural language processing (NLP) |
| Badal et al. (2021) | USA | Loneliness Detection | Survey on elderly individuals in the independent living area of a Continuing Care Senior Housing Community (CCSHC) in San Diego County, California, USA | 80 | SVM, kNN, DT, AdaBoost, ANN, RF, and Stack. | Predicting loneliness in the elderly based on interview text features extracted by NLP |
| Byeon (2021) | South Korea | Anxiety Prediction | Korean Psychosocial Anxiety (KPA) Survey | 1,558 | SVM, RF, LightGBM, AdaBoost, and XGBoost. | Identifying predictors of high-risk groups for anxiety disorders in the elderly |
| Byeon (2022) | South Korea | Depression Prediction | The 7th National Health and Nutrition Examination Survey | 582 | GBM, RF, AdaBoost, SVM, and XGBoost, Stack (GBM + BR), Stack (RF + BR), Stack (AdaBoost + BR), Stack (SVM + BR), Stack (XGBoost + BR), and Stack (GBM + RF + AdaBoost + SVM + XGBoost + BR). | Developing a prediction model for depression among elderly women living alone |
| Castillo-Hornero et al. (2024) | Spain | Loneliness Detection | Natural language texts collected via chatbots and online questionnaires on the Qualtrics platform | 1,112 | LR, and NB. | Identifying the level of loneliness in the elderly from natural language |
| Chen et al. (2022) | China | Suicide Prediction | Suicide death cases selected using two-stage stratified cluster sampling, with 1:1 age- and gender-matched living controls selected in the same region | 484 | CART. | Identifying risk factors for suicide in the elderly and analyzing the internal mechanism of the relationship between these factors and suicide risk |
| Chen et al. (2023) | China | Depression Prediction | 2011-2018 China Health and Retirement Longitudinal Survey (CHARLS) | 22,625 | DML. | Evaluating the effect of overage labor on depression in elderly rural residents |
| Chen et al. (2024) | China | Life Satisfaction Prediction | 2011-2018 China Health and Retirement Longitudinal Survey (CHARLS) | 2,600 | RF, XGBoost, SVM, kNN, and MLP. | Identifying predictors of life satisfaction trajectories in the elderly |
| Chen et al. (2025) | USA | Depression Detection | Recruited elderly drivers | 157 | XGBoost, and LR. | Identifying the depression status of the elderly through natural driving data |
| Cho et al. (2021) | South Korea | Suicide Prediction | The big data health screening cohort provided by the National Health Insurance Sharing Service | 48,047 | RF. | Developing a suicide prediction model for the elderly |
| Cruz et al. (2023) | France | Well-being Prediction | Survey of Health, Ageing, and Retirement in Europe (SHARE) | 37,991 | RF. | Ranking the importance of predictors of subjective well-being in the elderly |
| Diniz et al. (2016) | USA | Depression Prediction | Recruited elderly individuals with remitted late-life depression (LLD) and no history of major depressive disorder or other major mental illnesses | 75 | SVM. | Using machine learning to determine the accuracy of specific proteins in correctly distinguishing LLD patients from control participants |
| Fang et al. (2024) | Singapore | Well-being Prediction | Survey on elderly individuals at different locations in Singapore using convenience sampling | 300 | MCA, DT, and LR. | Exploring the association between lifelong learning and subjective well-being of the elderly in Singapore |
| Fang et al. (2024) | China | Comprehensive Mental Health Prediction | Street data from OpenStreetMap, street view data from Baidu Maps, and questionnaire surveys conducted with random sampling from 24 communities | 462 | DCNN. | Investigating the relationship between the built environment and the physical and mental health of elderly residents |
| Finze et al. (2024) | Germany | Well-being Detection | Recruitment from institutions where elderly individuals gather weekly for charity programs, mutual conversations, lectures, and games | 32 | SVM, LASSO, RF, and XGBoost. | Assessing the subjective well-being of the elderly based on natural speech |
| Grzenda et al. (2021) | USA | Depression Prediction | Two clinical trials on adults aged 60 and above with depressive disorders | 67 | SVM, RF, and LR. | Predicting treatment outcomes of late-life depression |
| Hatton et al. (2019) | UK | Depression Prediction | The "treatment-as-usual" arm of the CASPER (CollAborative care and active surveillance for Screen-Positive EldeRs with subthreshold depression) trial | 284 | XGBoost, and LR. | Predicting the likelihood of persistent depressive symptoms in the elderly after 12 months |
| Helbich et al. (2019) | China | Depression Prediction | Mental health survey conducted by Renmin University of China in Haidian District, Beijing, China from March to August 2011, street view images from Tencent Maps, and GlobeLand30 land cover data | 1,190 | FCN. | Extracting street view green spaces and blue spaces from street view data |
| Hong et al. (2025) | China | Depression Prediction | 2018 China Health and Retirement Longitudinal Survey (CHARLS) | 3,107 | LR, and XGBoost. | Establishing a model to predict the risk of depression in disabled elderly individuals |
| Huang (2021) | China | Depression Detection | Four public microexpression and emotion-related datasets | N/A | VGG-16. | Identifying late-life depression by extracting facial micro-expressions |
| Javeed et al. (2025) | Sweden | Depression Prediction | Swedish National Study on Aging and Care (SNAC) | 726 | Relief_Lasso_NN, LR, GNB, AdaBoost, kNN, DT, RF, and SVC. | Identifying risk factors for depression in the elderly |
| Jiang et al. (2025) | China | Social Isolation Prediction | 2015 and 2018 China Health and Retirement Longitudinal Survey (CHARLS) | 6,588 | LightGBM, LR, DT, SVM, RF, GBDT, and XGBoost. | Identifying predictors of social isolation in the elderly |
| Jin et al. (2024) | China | Depression Prediction | 2011-2020 data from the China Health and Retirement Longitudinal Survey (CHARLS) and corresponding annual urban equivalent sound pressure level (ESPL) time-series monitoring data in China | N/A | QRM, BARTM, and BCFM. | Examining the non-linear effect of urban noise pollution on the depression scores of the elderly population in China |
| Joshanloo (2025) | USA | Perceived Aging Prediction | Health and Retirement Study (HRS) | 3,279 | RF. | Evaluating the relative importance of potential predictors of self-perceived aging in the elderly |
| Joshanloo (2025) | UK | Perceived Aging Prediction | The eighth wave of the English Longitudinal Study of Aging (ELSA) | 7,172 | RF, and XGBoost. | Identifying important predictors of self-perceived aging (SPA) |
| Kang et al. (2025) | South Korea | Social Isolation Prediction | Recruitment from dementia relief centers and community service centers in Seoul, South Korea | 99 | LR, RF, GBM, and XGBoost. | Exploring factors associated with low social interaction frequency and high levels of loneliness |
| Kim et al. (2019) | South Korea | Depression Prediction | Elderly individuals living alone recruited through community health centers in South Korea | 56 | LR, DT, GB, and RF. | Predicting the classification of depression levels in elderly individuals living alone |
| Kim et al. (2024) | South Korea | Well-being Prediction | 2019 National Leisure Activity Survey | 1,769 | Linear Regression, Ridge, LASSO, SVM, DT, RF, XGBoost, and LightGBM. | Exploring leisure-related factors affecting the well-being of the elderly |
| Konig et al. (2021) | France | Comprehensive Mental Health Detection | Recruitment through the Memory Clinic at Claude Pompidou Institute, Nice University Hospital | 141 | SVR, and LASSO. | Extracting information from speech features to predict Neuropsychiatric Inventory (NPI) scores |
| Lee et al. (2022) | South Korea | Depression Detection Anxiety Detection | Recruitment from Ewha Womans University Mokdong Hospital | 352 | LR, SVM, RF, and GB. | Identifying depression and anxiety in the elderly using low-cost activity trackers and basic geriatric assessment scales |
| Lee et al. (2024) | South Korea | Depression Detection Anxiety Detection | A preliminary study conducted at Ewha Womans University Mokdong Hospital from May 2017 to May 2019 | 352 | CNN, LSTM, ResNet, MLP, and Grid Search. | Constructing an end-to-end hybrid input deep learning model to realize multi-label screening for late-life depression and anxiety |
| Lee et al. (2025) | South Korea | Suicide Prediction | 2014 and 2017 Elderly Status Survey | 19,536 | LASSO, SVM, DT, RF, MIA, Boosting, MLP, and Mullainathan-Spiess Ensemble. | Predicting high-risk suicide groups among the elderly |
| Li et al. (2024) | China | Depression Prediction | China Health and Retirement Longitudinal Survey (CHARLS) and Osteoarthritis Initiative (OAI) | 1,611 | LASSO, LR, DT, RF, and ANN. | Predicting the risk of depressive symptoms in patients with Knee Osteoarthritis (KOA) |
| Li et al. (2024) | China | Depression Prediction | Chinese General Social Survey (CGSS) | 1,408 | LR, and DDML. | Examining the heterogeneous effects of interpersonal communication in different relationships on the depressive mood of the elderly |
| Li et al. (2025) | China | Depression Prediction | National Health and Nutrition Examination Survey (NHANES) | 945 | LASSO, XGBoost, LR, RF, and SVM. | Predicting the risk of depressive symptoms in elderly individuals with cognitive impairment |
| Li et al. (2025) | China | Self-Neglect Prediction | Ma'anshan Healthy Ageing Cohort (MHAC) | 2,494 | DT, kNN, LR, RF, SVM, and XGBoost. | Predicting the risk of self-neglect in the elderly |
| Lin et al. (2022) | China | Depression Detection | Recruited participants and the speech dataset Oizys | 109 | DepAudioNet (1D-CNN + LSTM), and PT-Transformer (7Conv + 24Trans). | Constructing a model to screen and diagnose depression in elderly patients using speech data |
| Lin et al. (2022) | China | Depression Prediction | 2011-2015 China Health and Retirement Longitudinal Survey (CHARLS) | 2,548 | LSTM, GBDT, SVM, and RF. | Identifying risk factors for depression in the elderly |
| Lin et al. (2022) | China | Depression Prediction | 2011 and 2013 China Health and Retirement Longitudinal Survey (CHARLS) | 3,570 | LR, LASSO, Ridge, and RF. | Establishing a model to distinguish elderly individuals who will develop depression from non-depressed elderly individuals in future family settings |
| Lin et al. (2023) | China | Depression Prediction | Patients aged 60 and above recruited from psychiatric outpatient clinics | 77 | 3D-CNN. | Distinguishing elderly patients with late-life depression (LLD) from non-depressed elderly individuals based on resting-state fMRI signals of the brain |
| Lin et al. (2023) | China | Depression Prediction | 2011-2018 China Health and Retirement Longitudinal Survey (CHARLS) | 2,650 | GBDT, SVM, and RF. | Predicting the occurrence and development trajectory of depressive symptoms in community-dwelling elderly individuals over a 7-year period |
| Lin et al. (2024) | China | Loneliness Prediction | 2018 Chinese Longitudinal Healthy Longevity Survey | 16,000 | LR, Ridge, SVM, KNN, DT, RF and MLP. | Developing a prediction model for loneliness in the elderly |
| Lin et al. (2024) | China | Suicide Prediction | Recruited patients with late-stage depression | 83 | 3D-CNN. | Predicting suicidal tendencies from the complexity of certain brain regions in resting-state fMRI data |
| Liu and Liao (2024) | China | Comprehensive Mental Health Prediction | A 2023 survey on the well-being of urban residents in Chongqing | 1,185 | XGBoost. | Ranking the importance of urban neighborhood environmental factors affecting the mental health of urban elderly individuals |
| Liu et al. (2025) | China | Depression Prediction | 2020 China Health and Retirement Longitudinal Survey (CHARLS) | 4,322 | LASSO, LR, RF, GBM, kNN and NB. | Constructing a risk prediction model for depression in elderly individuals with functional disabilities |
| Maekawa et al. (2024) | Brazil | Anxiety Prediction | Dataset from randomized controlled trials | 536 | BN, LR, SGDClassifier, XGBoost, and RVM. | Identifying important predictors of recurrent anxiety symptoms |
| Maestro et al. (2023) | Sweden | Emotion Detection | Physiological data collected by wearable medical sensors and data collected through a smartphone app with an EMA questionnaire | 4 | SVM, RF, and MLP. | Automatically detecting and classifying the emotional states of the elderly |
| Meda et al. (2024) | Italy | Suicide Prediction | Waves 2 to 9 of The Survey of Health, Ageing and Retirement in Europe (SHARE) | 146 | RF. | Identifying key variables for predicting suicide deaths |
| Merritt et al. (2022) | USA | Emotion Detection | Participants recruited from Texas | 2,478 | RLR, RF, and SVM. | Predicting low mood and low energy states in the elderly using neurophysiological data |
| Na et al. (2022) | South Korea | Suicide Prediction | Korea Welfare Panel Study (KOWEPS) | 6,410 | RF. | Screening important risk factors for suicidal ideation in the elderly |
| Nath et al. (2022) | USA | Stress Detection | Recruited healthy elderly individuals aged 60 to 80 | 19 | LR, RF, kNN, SVM, and LSTM. | Distinguishing between stressed and non-stressed states in the elderly using a wearable physiological stress monitoring system |
| Nejadshamsi et al. (2025) | Canada | Depression Prediction | Recruitment via online and face-to-face methods | 4 | Ensemble Variants (n=240). | Predicting the classification of depression levels in the elderly |
| Nguyen et al. (2022) | South Korea | Depression Prediction | 2020 Community Health Survey of the Republic of Korea | 36,258 | DNN. | Constructing a prediction model for the depression status of the elderly population during the pandemic |
| Niu et al. (2024) | China | Anxiety Prediction | 2017-2018 Chinese Longitudinal Healthy Longevity Survey (CLHLS) | 2,427 | LASSO, and XGBoost. | Screening key variables using LASSO regression and introducing the XGBoost model to predict the risk of anxiety symptoms in elderly patients with abdominal obesity |
| Park et al. (2025) | South Korea | Depression Prediction | Korean Longitudinal Study of Aging (KLoSA) | 5,153 | DT, AdaBoost, RF, GBC, ET, XGBoost, LightGBM, LR, LDA, QDA, NB, and kNN. | Developing a machine learning model for predicting future depression |
| Perera et al. (2025) | USA | Life Satisfaction Prediction | Health and Retirement Study (HRS) | 12,046 | MERF. | Determining the relative importance of five life domains in explaining the overall life satisfaction of the elderly |
| Qin et al. (2024) | China | Depression Prediction | National Health and Nutrition Examination Survey (NHANES) | 1,538 | RF, XGBoost, ANN, and SVM. | Constructing a classification model for depressive symptoms in elderly patients with Obstructive Sleep Apnea (OSA) |
| Sarpdagi et al. (2025) | Turkey | Attitude Toward Death Prediction | A survey conducted in a province in Turkey from February 2024 to June 2024 | 467 | kNN, SVR, EN, LASSO, Ridge, RF, XGBoost, avNNet, MONMLP, NN, and QRNN. | Identifying key factors affecting the death attitude of the elderly |
| Shang et al. (2025) | China | Depression Prediction | National Health and Nutrition Examination Survey (NHANES) | 4,240 | LASSO, RF, LR, XGBoost, GNB, and GBDT. | Performing feature selection using LASSO regression and constructing prediction models with five machine learning algorithms |
| Sheng et al. (2024) | China | Depression Prediction | 2018 China Health and Retirement Longitudinal Survey (CHARLS) | 10,906 | SVM, GLM, kNN, RF, and CART. | Investigating the association between frequent interaction or contact with children and the incidence of late-life depression |
| Site et al. (2022) | Finland | Loneliness Detection | Indoor and outdoor sensors carried by volunteers in a nursing home for the elderly in Tampere, Finland | 5 | LR, SVM, RF, and XGBoost. | Classifying the loneliness level of users based on their indoor and outdoor mobility patterns |
| Smith et al. (2020) | USA | Depression Detection | Elderly individuals recruited from 3 senior communities in Iowa, USA | 46 | SVM. | Realizing binary classification prediction of depressive symptoms and scale score prediction in the elderly based on speech features |
| Solomonov et al. (2021) | USA | Depression Prediction | The COPE-D project, a two-center randomized controlled trial conducted at Weill Cornell Medicine and the University of California, San Francisco from December 2002 to November 2007 | 221 | RF. | Identifying important baseline characteristics of different depressive symptom trajectories |
| Song et al. (2025) | China | Depression Prediction | 2020 China Health and Retirement Longitudinal Survey (CHARLS) | 7,880 | LR, kNN, SVM, DT, LightGBM, and RF. | Identifying risk factors for depression in the elderly |
| Su et al. (2021) | China | Depression Prediction | Waves 3-7 of the Chinese Longitudinal Healthy Longevity Survey (CLHLS) | 1,538 | LSTM, LR, LASSO, RF, GBDT, SVM, and DNN. | Identifying predictors of depression in the elderly |
| Susanty et al. (2023) | Indonesia | Depression Prediction | Dataset collected from 15 Community Health Centers (CHCs) in Kendari, Indonesia using a cross-sectional design | 1,252 | LR, RF, GBM, and DI-VNN. | Developing an alternative triage test for the 15-item Geriatric Depression Scale (GDS-15) in community-dwelling elderly individuals |
| Trescato et al. (2023) | Italy | Well-being Prediction | Waves 4 to 7 of The Survey of Health, Ageing and Retirement in Europe (SHARE) | 9,422 | LR, LASSO and RF. | Predicting changes in subjective well-being of the elderly over a two-year period |
| Wang and Yao (2025) | China | Depression Prediction | 2010 Shanghai regional data from the World Health Organization (WHO)’s “Study on Global AGEing and Adult Health (SAGE)” and street view data from Tencent Maps | 2,969 | FCN-8s, and RF. | Quantifying the quantity and quality of visual green spaces |
| Wang et al. (2019) | China | Comprehensive Mental Health Prediction | A 2011 mental health survey on the elderly in Beijing, China, and street view images from Tencent Maps | 1,231 | FCN. | Performing semantic segmentation on street view images |
| Wang et al. (2023) | China | Depression Prediction | 2014 China Longitudinal Ageing Social Survey | 7,496 | RF. | Analyzing the causal effects of physical disability and the number of comorbid chronic diseases on depressive symptoms |
| Wang et al. (2025) | China | Depression Prediction | 2018 China Health and Retirement Longitudinal Survey (CHARLS) | 1,269 | NN, AdaBoost, EN, GBR, LASSO, Ridge, and RF. | Identifying risk factors for depression in the elderly |
| Wu et al. (2023) | China | Depression Prediction | 2016, 2018 and 2020 China Family Panel Studies (CFPS) | 20,720 | RF. | Identifying predictors of depression in the elderly |
| Xin et al. (2022) | China | Depression Prediction | China Family Panel Studies (CFPS) | 1,460 | RF. | Predicting depression in disabled elderly individuals from multiple aspects |
| Xiong et al. (2025) | China | Suicide Prediction | Elderly inpatients with non-psychiatric diseases at Guangdong Provincial Hospital of Traditional Chinese Medicine | 807 | LR, and RF. | Establishing a prediction model for suicidal ideation in elderly inpatients without psychiatric diseases |
| Xu et al. (2019) | USA | Depression Prediction | The 22-year longitudinal survey data from the University of Michigan Health and Retirement Study | 5,686 | LSTM, Multitask LSTM, MLP Classifier, SVM, DBN, MLP Regressor, and LASSO-GLM. | Developing a deep learning model for individualized prediction of depressive disorders |
| Yildiz et al. (2023) | Turkey | Depression Prediction | Elderly individuals from the Health Bureau of an agricultural province | 319 | LR, and RF. | Determining the correlation between elderly adaptation, home-based care, and depression |
| Zhang et al. (2025) | China | Comprehensive Mental Health Prediction | Elderly individuals recruited in Nanjing, China | 1,007 | XGBoost. | Evaluating the relative importance of multiple green space indicators in predicting the mental health of the elderly |
| Zheng et al. (2024) | China | Depression Prediction | 2018 China Health and Retirement Longitudinal Survey (CHARLS) | 3,959 | LR, AdaBoost, RF, kNN, and SVM. | Identifying key predictors of depression in elderly individuals with chronic diseases |
| Zheng et al. (2024) | China | Depression Prediction | 2011-2020 China Health and Retirement Longitudinal Survey (CHARLS) | 11,342 | LR, AdaBoost, RF, and kNN. | Identifying predictors of depressive symptoms in elderly individuals with chronic diseases within 2 years |
| Zhou et al. (2023) | China | Emotion Detection | Elderly individuals diagnosed with mild cognitive impairment in a tertiary hospital | 319 | RF. | Developing a multi-class emotion classification model |
| Zhu et al. (2024) | China | Comprehensive Mental Health Prediction | 2014 China Longitudinal Aging Social Survey (CLASS) | 4,512 | CFM. | Estimating the heterogeneity of the relationship between employment and mental health |
| Zhu et al. (2025) | China | Loneliness Prediction | 2016, 2018 and 2020 China Family Panel Studies (CFPS) | 20,675 | RF. | Identifying predictors of loneliness in the elderly |
| Xu et al. (2025) | China | Depression Prediction | 2015 and 2018 China Health and Retirement Longitudinal Study (CHARLS) | 1,921 | GBM, RF, and XGBoost | Predicting the risk of depression within three years among older adults with subjective cognitive decline (SCD) |
| Zhang et al. (2026) | China | Depression Prediction | 2018 China Health and Retirement Longitudinal Study (CHARLS) | 16,627 | RF, and CFM | Analyzing the main influencing factors of depression in older adults and evaluating intervention programs for older adults with comorbid depression |
| Yadav and Bhutia (2025) | India | Depression Prediction | Wave 1 data from the Longitudinal Ageing Study in India (LASI) | 58,467 | RF, DT, LR, SVM, kNN, NB, NN, and Ridge | Identifying predictors of depression in older adults |
| Fang et al. (2025) | China | Depression Prediction | Five waves of the China Health and Retirement Longitudinal Study (CHARLS) | 3,819 | RF, DT, XGBoost, and SVM | Predicting trajectory types of depressive symptoms in older adults |
| Dogan and Hacikoylu (2026) | Turkey | Death Anxiety Prediction | Cross-sectional study conducted at a teaching and research hospital of a university in the central Black Sea region of Turkey | 169 | ANN | Identifying demographic characteristics that predict attitudes toward aging and old age and death anxiety among older adults with chronic diseases |
| Xu et al. (2025) | China | Depression Prediction | 2011 Global Burden of Disease, Injuries, and Risk Factors Study (GBD) data, China Health and Retirement Longitudinal Study (CHARLS) data (2015, 2018, and 2020), and National Health and Nutrition Examination Survey (NHANES) data (2013–2020) | 79,123 | XGBoost, and GA | Identifying key predictors of depression among older adult populations in China and the United States |
| Xu et al. (2026) | China | Well-being Prediction | Questionnaire survey conducted among elderly people in Nanjing, China | 536 | DT, RF, and ANN | Identifying key predictors of well-being in older adults |
| Liu et al. (2025) | China | Kinesiophobia Prediction | Elderly patients with chronic low back pain admitted to spinal surgery and orthopedic rehabilitation centers between January and December 2024 | 386 | LR, DT, RF, XGBoost, LightGBM, and ANN | Identifying key factors influencing kinesiophobia in older adults with chronic low back pain |
| Seo et al. (2025) | South Korea | Depression Prediction Anxiety Prediction | 2013, 2015, 2017, and 2019 Korea National Health and Nutrition Examination Survey (KNHANES) data | 5,419 | LR, SVM, DT, RF, and XGBoost | Identifying predictors of depression and suicidal ideation in older adults |
| Xilatu et al. (2025) | China | Life Satisfaction Prediction | A survey on digital inclusion among older adults conducted in three Chinese cities (Chongqing, Wuhan, and Shanghai) | 1,102 | RLR, RF, XGBoost, and SVM | Identifying predictors of life satisfaction in older adults |
| Long et al. (2026) | China | Depression Prediction | Five waves of the China Health and Retirement Longitudinal Study (CHARLS) (2011–2020) and the Chinese Longitudinal Healthy Longevity Survey (CLHLS) (2008–2018) | 4,700 | LR, LDA, QDA, GNB, DT, RF, ET, GBM, LightGBM, XGBoost, AdaBoost, kNN, SVM, LSTM, and DARNet | Identifying predictors of depression in older adults |
| Wang and Pan (2026) | China | Depression Prediction | 2018 China Health and Retirement Longitudinal Study (CHARLS) | 3,232 | LASSO, SVM, CNN, DT, XGBoost, RF, and LR | Identifying predictors of depression in rural older adults |
| An et al. (2025) | China | Depression Prediction | 2011 and 2015 China Health and Retirement Longitudinal Study (CHARLS) | 1,154 | LASSO, EN, CART, NB, PLS, kNN, RF, glmBoost, and CART | Identifying predictors of depression in older adults with asthma |
| Lin et al. (2026) | China | Suicide Prediction | Patients with late-life depression (LLD) recruited from the Brain Hospital of Guangzhou Medical University and normal controls (NC) from the community | 96 | SVM | Identifying key brain network features predicting suicidal ideation in older adults with depression |
| Zhang et al. (2025) | China | Depression Prediction | Participants recruited from the Neurology Department and Physical Examination Center of Zhongnan Hospital of Wuhan University, and Shuiguohu Street Community Health Service Center | 602 | LightGBM, XGBoost, RF, AdaBoost, Bagging, and ET | Identifying clinical, imaging, and biomarker predictors of vascular depression (VaDep) |
| Zhang et al. (2026) | China | Suicide Prediction | Inpatients recruited from a tertiary hospital in China | 516 | LR, kNN, SVM, XGBoost, LightGBM, GBDT, RF, Stack, and GNB | Identifying predictors of suicidal ideation in older adults with chronic pain |
| Xu et al. (2025) | China | Self-Compassion Prediction | Elderly individuals with type 2 diabetes recruited in Jiaxing, China | 298 | RF, SVM, kNN, XGBoost, DT, and NB | Identifying factors contributing to self-compassion in community-dwelling older adults with type 2 diabetes |
| Lin et al. (2025) | China | Depression Detection | Elderly individuals recruited in Xiamen, China | 92 | LR, SVM, GBDT, and RF | Developing a gait-based depression recognition method |
| Qiu and Zhu (2026) | China | Depression Prediction | Waves 3 and 4 of the China Health and Retirement Longitudinal Study (CHARLS) | 5,310 | kNN, GNB, DT, RF, and XGBoost | Predicting future depressive status in older adults and assessing whether currently depressed older adults will achieve remission in the future |
| Mao et al. (2026) | China | Depression Prediction | Representative data collected from elderly populations across 16 urban districts of Beijing, China (covering both urban and rural areas) between 2022 and 2023 | 14,049 | SVM, RF, XGBoost, and LR | Identifying predictors of depression in older adults |
| Kong et al. (2025) | China | Life Satisfaction Prediction | Digital Divide Survey conducted by the National Information Society Agency (NIA) of South Korea between September and December 2023 | 869 | RF, XGBoost, LightGBM, and CatBoost | Identifying key digital divide factors significantly associated with life satisfaction |
| Kim et al. (2025) | South Korea | Suicide Prediction | Individuals participating in the Bus-based Screening and Assessment Network (BUSAN) project from July 1 to December 31, 2023 | 238 | SVM, RF, LR, LDA, and GB | Identifying predictors of suicidal ideation in older adults |
| Wang et al. (2026) | China | Depression Prediction | 2011–2018 National Health and Nutrition Examination Survey (NHANES) | 3,802 | BNB, GNB, kNN, LR, RF, SVM, and XGBoost | Identifying predictors of depression in older adults |
| Murri et al. (2025) | Italy | Depression Prediction | Waves 5–8 of the Survey of Health, Ageing and Retirement in Europe (SHARE) | 1,360 | DT, RF, and GLM | Developing a risk prediction model for depression in older cancer patients |
| Takemura et al. (2025) | Japan | Depression Prediction | 2016, 2019, and 2022 waves of the Japan Gerontological Evaluation Study (JAGES) | 11,146 | CFM | Identifying which older adults benefit most from social engagement in terms of greater reduction in depression risk |
| Aydin et al. (2025) | Turkey | Quality of Life Prediction | Sampling survey of elderly people residing in Erzurum | 451 | kNN, SVM, avNNet, MONMLP, pcaNNet, RF, XGBoost, and xgbLinear | Identifying predictors of quality of life in older adults |
| Wang et al. (2026) | China | Depression Prediction | 2011, 2015 and 2020 China Health and Retirement Longitudinal Study (CHARLS) | 18,980 | RF, and LR | Using random forest models to assess important factors predicting depressive symptoms and their changes over time, and logistic regression to estimate the direction and magnitude of these associations |
| Liu et al. (2026) | China | Depression Prediction | Health and Retirement Study (HRS) and China Health and Retirement Longitudinal Study (CHARLS) | 11,341 | GBM, XGBoost, LR, SVM, NN, GBM, NB, LDA, kNN, CART, AdaBoost, LASSO, MARS, DT, PLS, CIT, and GLM | Identifying predictors of depression in older adults |
| Guo et al. (2025) | China | Well-being Prediction | A survey on living environments and mental health among older adults (aged 60–75) conducted between October and December 2023, and street view images from Baidu Maps | 250 | DeeplabV3 + ResNet101, and RF | Using DeepLabV3 + ResNet101 for image semantic segmentation to extract tree morphological features, and random forest to identify predictors of well-being in older adults |
| Lee and Lee (2026) | South Korea | Suicide Prediction | Korean Elderly Survey conducted by the Ministry of Health and Welfare of the Republic of Korea in 2014 and 2017 | 19536 | DT, RF, ET, AdaBoost, GBDT, LightGBM, MLP | Identify risk factors from suicidal ideation to suicide attempt among the elderly |
| Zhang et al. (2026) | China | Depression Prediction | Elderly stroke patients admitted to Xiangyang Central Hospital between January 2016 and December 2023 | 691 | LR, RF, SVM, XGBoost, LightGBM, AdaBoost, MLP | Identify risk factors for post-stroke depression (PSD) in the elderly |
| Bello-Valle et al. (2022) | Mexico | Loneliness Prediction Social Isolation Prediction | Loneliness and Social Isolation Datasets | 244 | DT, CART, SVM, AdaBoost_RF, kNN, NB. | Constructing a joint prediction model for loneliness and social isolation |
| Liu et al. (2023) | China | Comprehensive Mental Health Prediction | Data from the 2019 "Healthy Aging - Elderly Psychological Care 13th Five-Year Plan" project, NHC of China | 15,173 | RF, XGBoost, LightGBM. | Developing a predictive model for mental health issues among older adults |
| Wang et al. (2026) | China | Depression Prediction | 2018 China Health and Retirement Longitudinal Study (CHARLS) | 5,564 | kNN, LightGBM, NN, SVM, XGBoost. | Determining predictors of depressive symptoms in older individuals with chronic diseases |
| Xian et al. (2026) | China | Anxiety Prediction | 2018 Chinese Longitudinal Healthy Longevity Survey (CLHLS) | 9,535 | RF, XGBoost, LightGBM, DT, LR. | Determining predictors of anxiety symptoms in older adults |
| Bae et al. (2025) | South Korea | Life Satisfaction Prediction | The 2020 Korea Senior Survey | 1,411 | LASSO, CART, DT, RF, XGBoost. | Identifying predictors of life satisfaction among older adults living alone |
| Imans et al. (2024) | South Korea | Depression Detection Depression Prediction | 2005–2006 National Social Life, Health, and Aging Project (NSHAP) data | 2,763 | DT, LR, NB, kNN, MLP, SVC, RF, XGBoost, GB, AdaBoost, CatBoost, LightGBM, Vot, DES methods, FIRE-DES. | Detecting the presence of depression and predicting its severity |
| Mu et al. (2026) | USA | Comprehensive Mental Health Detection | The Internet-Based Conversational Engagement Clinical Trial (I-CONECT) study | 39 | LR, GBDT, SVM, RF, HMM, LSTM, Transformer, MLP. | Using HMM for temporal encoding and other models for psychological state classification |
| Guo et al. (2024) | China | Depression Prediction | Older adults recruited from Shanghai, China | 368 | BN, NB, NBM, LR, MLP, SGD, SVM, kNN, AdaBoost, Bagging, Ensemble Optimizer, DT, RF. | Establishing a predictive model for depression in patients with hypertension |
| Jung et al. (2022) | South Korea | Depression Detection | Community-dwelling older adults aged 65 and above | 45 | BiLSTM. | Identifying depression in older adults |
| Kim et al. (2023) | South Korea | Suicide Prediction | Older adults recruited from rural areas of South Korea | 650 | DT, RF, LR. | Identifying risk factors for suicidal ideation |
| Chen et al. (2023) | China | Psychological Resilience Prediction | Urban and rural older adults in a city of Taiwan, China | 936 | RF, GB, SVM, LR. | Detecting and ranking key factors influencing psychological resilience in older adults during COVID-19 |
| Lin et al. (2023) | China | Suicide Detection | LLD patients from the Affiliated Brain Hospital of Guangzhou Medical University and healthy controls from the local community | 127 | SVM. | Exploring abnormal functional connectivity in the dlPFC network in late-life depression with suicidal ideation |
| Wang et al. (2025) | China | Depression Prediction | 2020 China Health and Retirement Longitudinal Study (CHARLS) data | 17,424 | LCA, LR, XGBoost. | Identifying comorbidity patterns that predict depression |
| Aswathy et al. (2025) | India | Depression Prediction | Wave 1 of the Longitudinal Ageing Study in India (LASI) | 26,065 | XGBoost. | Evaluating the effectiveness of physiological, psychological, and functional health factors in classifying depressive symptom levels |
| Choi et al. (2018) | USA | Depression Prediction | Wave 2 of the National Social Life, Health, and Aging Project (NSHAP), USA | 3,377 | LASSO, LR. | Constructing a predictive model for depression |
| Xiang et al. (2025) | USA | Psychological Resilience Prediction | 2016 and 2020 Health and Retirement Study (HRS) data | 3,364 | LASSO, Ridge, RF. | Identifying predictors of psychological resilience in older adults during the COVID-19 pandemic |
| Byeon (2021) | South Korea | Depression Prediction | 2016 Seoul Panel Study (SEPANS) | 4,085 | LR, GBM, RF. | Establishing a predictive model for depression among community-dwelling older adults |
| Jin and Halili (2025) | China | Depression Prediction | 2011–2015 China Health and Retirement Longitudinal Study (CHARLS) data | 5,383 | LASSO, EN, Boruta, LR, HistGBM, MLP, XGBoost, Bagging, DT, LightGBM, RF, SVM, CatBoost. | Identifying risk factors for depression in older adults with disabilities |
| Shen et al. (2023) | China | Life Satisfaction Prediction | Health and Retirement Study (HRS) Waves 10–14 (2010–2018) | 34,630 | SVR, MLR, Ridge, LASSO, kNN, DT. | Identifying predictors of life satisfaction in older adults |
| Paul et al. (2025) | Germany | Quality of Life Prediction | Epidemiological study on potentials for prevention, early detection and optimized treatment of chronic diseases in the elderly population (ESTHER) | 3,124 | RF. | Identifying predictors of Health-Related Quality of Life (HRQoL) |
| Oken et al. (2018) | USA | Comprehensive Mental Health Prediction | A randomized controlled clinical trial conducted in the Portland metropolitan area, Oregon | 134 | DT. | Predicting individuals most likely to achieve mental health improvements from mindfulness meditation |
| Georgescu et al. (2024) | UK | Depression Detection Anxiety Detection | Older participants recruited through the Laços Saúde company | 69 | RF. | Screening and classifying symptoms of depression and anxiety in older adults |
| Chen et al. (2026) | China | Depression Prediction | Survey data of older adults in Guangzhou, China | 2,585 | LR, SVM, RF, XGBoost. | Identifying predictors of depression in older adults |
| Jin et al. (2021) | China | Depression Prediction | Two waves of The Irish Longitudinal Study on Ageing (TILDA) | 6,715 | RF. | Identifying predictors of depression in older adults |
| Shiba et al. (2022) | USA | Comprehensive Mental Health Prediction | Japan Gerontological Evaluation Study (JAGES) | 3,567 | RF. | Estimating conditional average treatment effects of disaster damage on post-disaster mental health outcomes |
| Asadollahi et al. (2026) | Iran | Comprehensive Mental Health Prediction | Sample questionnaire survey and municipal environment database in Shiraz, Iran | 3,000 | DT, SVM. | Identifying predictors influencing health outcomes in older adults |
| Moore et al. (2025) | USA | Well-being Prediction | Survey of Health, Ageing and Retirement in Europe (SHARE) | 37,991 | RF. | Screen variables and determine the ranking results of predictive factors |
| Zhao et al. (2026) | China | Life Satisfaction Prediction | 2014 and 2020 China Health and Retirement Longitudinal Study (CHARLS) data | 4,627 | LR, XGBoost. | Identifying key determinants for different levels of life satisfaction |

*Notes: "3D-CNN" = "Three-Dimensional Convolutional Neural Network", "AdaBoost" = "Adaptive Boosting", "AdaBoost_RF" = "AdaBoost with Random Forest classifier", "ANN" = "Artificial Neural Network", "avNNet" = "Model Averaged Neural Network", "Bagging" = "Bootstrap Aggregating", "BARTM" = "Bayesian Additive Regression Tree Model", "BCFM" = "Bayesian Causal Forest Method", "BiLSTM" = "Bidirectional Long Short-Term Memory", "BN" = "Bayesian Network", "BNB" = "Bernoulli Naive Bayes", "Boruta" = "Boruta Feature Selection", "BR" = "Bayesian Regression", "CART" = "Classification and Regression Tree", "CatBoost" = "Categorical Boosting", "CFM" = "Causal Forest Model", "CIT" = "Conditional Inference Tree", "CNN" = "Convolutional Neural Network", "DARNet" = "Dual Attention Residual Network", "DBN" = "Dynamic Bayesian Network", "DCNN" = "Deep Convolutional Neural Network", "DDML" = "Double Debiased Machine Learning", "DepAudioNet (1D-CNN + LSTM)" = "DepAudioNet (1D Convolutional Neural Network + Long Short-Term Memory)", "DES methods" = "Dynamic Ensemble Selection (KNORAE, METADES, etc.)", "DI-VNN" = "Deep Insight Visible Neural Network", "DML" = "Double Machine Learning", "DNN" = "Deep Neural Network", "DT" = "Decision Tree", "EN" = "Elastic Net", "Ensemble Optimizer" = "Filtered Classifier / Iterative Classifier Optimizer", "ET" = "Extra Trees Classifier", "FCN" = "Fully Convolutional Network", "FCN-8s" = "Fully Convolutional Network with 8s skip connections", "FIRE-DES" = "Fuzzy Information-based DES", "GA" = "Genetic Algorithm", "GB" = "Gradient Boosting", "GBC" = "Gradient Boosting Classifier", "GBDT" = "Gradient Boosting Decision Tree", "GBM" = "Gradient Boosting Machine", "GBR" = "Gradient Boosting Regression", "GLM" = "Generalized Linear Model", "glmBoost" = "Gradient Boosting with Component-wise Linear Models", "GNB" = "Gaussian Naive Bayes", "HistGBM" = "Histogram-based Gradient Boosting Machine", "HMM" = "Hidden Markov Model", "kNN" = "k-Nearest Neighbor", "LASSO" = "Least Absolute Shrinkage and Selection Operator", "LASSO-GLM" = "LASSO Generalized Linear Model", "LCA" = "Latent Class Analysis", "LDA" = "Linear Discriminant Analysis", "LightGBM" = "Light Gradient Boosting Machine", "LR" = "Logistic Regression", "LSTM" = "Long Short-Term Memory", "MARS" = "Multivariate Adaptive Regression Splines", "MCA" = "Multiple Correspondence Analysis", "MERF" = "Mixed Effects Random Forest", "MIA" = "Missing Incorporated in Attributes Random Forest", "MLP" = "Multi-Layer Perceptron", "MLP Classifier" = "Multi-Layer Perceptron Classifier", "MLP Regressor" = "Multi-Layer Perceptron Regressor", "MLR" = "Multiple Linear Regression", "MONMLP" = "Monotone Multi-Layer Perceptron", "Mullainathan-Spiess Ensemble" = "Inspired Ensemble Method", "Multitask LSTM" = "Multitask Long Short-Term Memory Network", "NB" = "Naive Bayes", "NBM" = "NaiveBayesMultinomial", "NN" = "Neural Network", "pcaNNet" = "Neural Networks with a Principal Component Step", "PLS" = "Partial Least Squares", "PT-Transformer (7Conv + 24Trans)" = "Pre-trained Transformer (7 Convolutional Layers + 24 Transformer Blocks)", "QDA" = "Quadratic Discriminant Analysis", "QRM" = "Quantile Regression Model", "QRNN" = "Quantile Regression Neural Network", "Relief_Lasso_NN" = "Integrated Relief_Lasso feature selection with Neural Network", "ResNet" = "Residual Network", "RF" = "Random Forest", "Ridge" = "Ridge Regression", "RLR" = "Regularized Logistic Regression", "RVM" = "Relevance Vector Machine", "SGD" = "Stochastic Gradient Descent", "SGDClassifier" = "Stochastic Gradient Descent Classifier", "Stack" = "Stacking Ensemble", "SVC" = "Support Vector Classification", "SVM" = "Support Vector Machine", "SVR" = "Support Vector Regression", "Transformer" = "Transformer Network", "VGG-16" = "Visual Geometry Group 16-layer Convolutional Neural Network", "Vot" = "Voting Classifier", "XGBoost" = "eXtreme Gradient Boosting", "xgbLinear" = "XGBoost with Linear Booster".*
